# Impact of impaired glomerular filtration rate and revascularization strategy on one-year cardiovascular events in acute coronary syndrome: data from Taiwan acute coronary syndrome full spectrum registry

**DOI:** 10.1186/1471-2369-15-66

**Published:** 2014-04-23

**Authors:** Tsung-Hsien Lin, Ho-Tsung Hsin, Chun-Li Wang, Wen-Ter Lai, Ai-Hsien Li, Chi-Tai Kuo, Juey-Jen Hwang, Fu-Tien Chiang, Shu-Chen Chang, Chee-Jen Chang

**Affiliations:** 1 Division of Cardiology, Department of Internal Medicine, Kaohsiung Medical University Hospital, No.100, Tzyou 1st Road, Kaohsiung 80708, Taiwan; 2 Department of Internal Medicine, Faculty of Medicine, Kaohsiung Medical University, Kaohsiung, Taiwan; 3 Division of Cardiology, Department of Internal Medicine, Far Eastern Memorial Hospital, Taipei, Taiwan; 4 Chang Gung University College of Medicine, Taoyuan, Taiwan; 5 Division of Cardiology, Department of Internal Medicine, Linkou Chang Gung Memorial Hospital, Linkou, Taiwan; 6 Division of Cardiology, Department of Internal Medicine, National Taiwan University Hospital, Taipei, Taiwan; 7 Division of Biostatistics, Institute of Public Health, National Yang-Ming University, Taipei, Taiwan; 8 Graduate Institute of Clinical Medicine, Research Center for Clinical Informatics and Medical Statistics, Chang Gung University, Taoyuan, Taiwan

**Keywords:** Acute coronary syndrome, Chronic kidney disease, Invasive, Revascularization, Angiography

## Abstract

**Background:**

The optimal revascularization strategy for patients with impaired glomerular filtration rate (IGFR) has not been established in acute coronary syndrome (ACS). We investigated the prognosis and impact of IGFR and invasive strategy on the cardiovascular outcomes in the ACS population.

**Methods:**

In a Taiwan national-wide registry, 3093 ACS patients were enrolled. The invasive strategy was defined as patients with ST-elevation ACS (STE-ACS) undergoing primary angioplasty or fibrinolysis or coronary angiography with intent to revascularization performed within 72 hours of symptom onset in non-ST-elevation ACS (NSTE-ACS). IGFR was defined as an estimated GFR of less than 60 ml/min per 1.73 m^2^. Primary endpoint was a composite of death, non-fatal myocardial infarction or stroke at one year.

**Results:**

Patients with IGFR (n = 1226) had more comorbidities but received less evidence-based medications during admission than those without IGFR (n = 1867). The primary endpoint-free survival rate is lower in the IGFR patients, in the whole, STE-ACS and NSTE-ACS population (all log-rank tests p < 0.01). Cox regression analysis revealed IGFR subjects had higher primary endpoint after adjusting by age, sex, medication at discharge and traditional risk factors (all p < 0.01). Kaplan–Meier curves showed IGFR patients without invasive strategy had the worst outcome in the STE-ACS and NSTE-ACS population (both p < 0.01). The invasive strategies, either with early angiography only or angioplasty, were associated with reduced primary endpoints among IGFR patients in the NSTE-ACS population (both p ≦ 0.024).

**Conclusions:**

IGFR patients suffering from ACS had poor prognosis and an invasive strategy could improve cardiovascular outcome in the NSTE-ACS population.

## Background

Chronic kidney disease (CKD) is a global problem [1]. Patients with CKD have higher risk of progression to end-stage renal disease (ESRD) and poor cardiovascular prognosis [2]. Taiwan has been recognized as an epidemic area of kidney disease with the highest incidence and prevalence rates of ESRD in the world [3]. Although the nationwide CKD Preventive Project with multidisciplinary care program has proved its effectiveness in decreasing dialysis incidence, mortality and medical costs, the number of patients with CKD is still growing due to the increasing prevalence of comorbidities, such as hypertension and diabetes, in Taiwan as well as in the worldwide [4]. Therefore, the development of effective treatment strategies is mandatory for such a high cardiovascular risk population.

The presence of endothelial dysfunction, inflammation, dyslipidemia and activation of the renin-angiotensin system are the main mechanisms by which CKD can induce or complicate cardiovascular disease [5]. Therefore, CKD is not only a coronary risk equivalent for ascertainment of coronary risk but also a risk factor for the development and progression of cardiovascular disease (CVD) [6]. Furthermore cardiovascular death is the leading cause of mortality in the CKD population. Among the patients admitted due to acute coronary syndrome (ACS), those with CKD consistently have a poorer prognosis [7,8]. Although recent major clinical trials have shown aggressive medication treatment can improve cardiovascular outcome in patients suffering from ACS, only few patients with impaired glomerular filtration rate (IGFR) were enrolled.

In addition to the medical treatment, coronary revascularization such as percutaneous coronary intervention (PCI) is also proven to reduce cardiovascular morbidity and mortality in the ACS patients. However, limited studies investigated the impact of an invasive strategy on clinical endpoints in patients with ACS and IGFR, as many trials of revascularization excluded patients with IGFR [9]. Furthermore, with some evidence support, many physicians hesitate to perform coronary angiography and revascularization in IGFR patients because of the possibility of contrast-induced nephropathy.

The current analysis from Taiwan ACS Full Spectrum Registry therefore focuses on the prognosis and cardiovascular outcomes of invasive strategy in ACS patients with IGFR, including primary angioplasty or fibrinolysis for ST-elevation (STE-ACS) and early invasive strategy (EIS) for non-ST-elevation patients (NSTE-ACS) [10].

## Methods

### Study design

The study was a prospective, national, multicenter, non-interventional, observational design. Patients recruitment, definition of ACS, inclusion and exclusion had been previously described in detail [11]. Patient data, such as baseline characteristics, risk factors, clinical presentation, clinical diagnosis, in-hospital interventions as well as medications prescribed, were collected from admission to discharge. Patients were followed up at months 3, 6, 9 and 12 post-discharge and data was collected on medication usage, revascularization strategy as well as clinical events, like death, myocardial infarction, stroke, revascularization and hospitalization. Monitoring for source documentation and accuracy was performed in 5% of all case report forms at each recruiting site. This study was carried out in accordance with the local regulatory guidelines and international guidelines for Good Epidemiological Practice [12]. Ethics committee approval was obtained at all trial sites including China University Medical Hospital, Taoyuan General Hospital, Wan-Fang Hospital, Show Chwan Memorial Hospital, Chia-Yi Christian Hospital, Kuang Tien General Hospital, National Taiwan University Hospital, Cheng Ching Hospital, Sin Lau Hospital The Presbyterian Church of Taiwan, Tainan Municipal Hospital, Mackay Memorial Hospital, E-Da Hospital, Chi-Mei Hospital, Taichung Armed Forces General Hospital, Taipei Tzu Chi General Hospital, Kaohsiung Medical University Chung-Ho Memorial Hospital, Taichung Veterans General Hospital, Pingtung Christian Hospital, Lo-Tung Po-Ai Hospital, Far Eastern Memorial Hospital, National Cheng Kung University Hospital, National Taiwan University Hospital, Yun-lin Branch, Dalin Tzuchi General Hospital, Kee-lung Hospital, Taipei Veterans General Hospital, Cathay General Hospital, Kaohsiung Veterans General Hospital, Taipei Medical University Hospital, Shin Kong Wu Ho-Su Memorial Hospital, Changhua Christian Hospital, National Taiwan University Hospital, Chung Shan Medical University Hospita, Hualien Tzu Chi General Hospital, Mackay Memorial Hospital, Taitung Branch, Linkou Chang Gung Memorial Hospital, Hsin Chu General Hospital, Kaohsiung Chang Gung Memorial Hospital, Tri-Service General Hospital and Cheng-Hsin Hospital. Written informed consent was obtained from each patient.

Invasive strategy was defined as reperfusion done either by primary angioplasty or thrombolysis in STE-ACS or diagnostic coronary angiography (DCA) with intent to revascularization performed within 72 hours of symptom onset as early invasive strategy in NSTE-ACS.

### Calculation of kidney function and definition of IGFR

Creatinine was analyzed by the Jaffe-kinetic method in the central laboratory of each hospital. Baseline creatinine was defined as creatinine measurement at time of presentation. The estimated glomerular filtration rate (eGFR) was calculated using the Chronic Kidney Disease Epidemiology Collaboration equation [13]. IGFR was defined as a eGFR less than 60 ml/min per 1.73 m^2^. This range corresponds to stage 3 or higher CKD by the National Kidney Foundation’s classification scheme and helps identify individuals with clinically significant CKD [14].

### Statistical analyses

All data were expressed as mean ± standard deviation (SD). For comparability between groups, a chi-square test was used for categorical variables and analysis of variance (ANOVA) was adopted for continuous variables. One-year survival analysis was performed and the time to event was estimated according to the Kaplan-Meier method and compared using the log-rank test. Cox regression analysis was conducted to calculate hazard ratio for CVD after adjusting co-variables including age, sex and evidence-based medicines at discharge (aspirin, clopidogrel, ACE inhibitor, angiotensin II receptor blocker, oral b-blocker and statin), dyslipidemia, hypertension, diabetes, smoking and family history. Analyses were conducted as time to first event without double counting of events within analyses involving composite endpoints.

The primary outcome was the composite of death, non-fatal myocardial infarction and non-fatal stroke at one year. The secondary outcome was the composite of death, non-fatal myocardial infarction, non-fatal stroke, re-hospitalization and revascularization at one year. We analyzed the whole, STE-ACS and NSTE-ACS populations separately. Statistical analysis was performed using SAS software version 9.2 (SAS Institute Inc., Cary, NC, USA). All statistical analyses were performed using a level of < 0.05 with two-sided testing and this was considered as statistically significant.

## Results

### Clinical characteristics

A total of 3183 eligible patients were enrolled between October 2008 and January 2010 at 39 medical centers and regional hospitals in Taiwan [9]. Among them, 3093 (97.1%) subjects with renal parameters and 12 months outcome data were analyzed in this study and 1631 (52.7%) patients were STE-ACS. The mean age of the 3093 patients was 63.11 ± 13.50 years old and 78.2% of the patients were males.

Baseline creatinines were 2.74 ± 2.59 and 0.94 ± 0.18 mg/dl in the IGFR (n = 1226) and non-IGFR (n = 1867) groups, respectively. Compared with the non-IGFR patients, those with IGFR were older, thinner, lower diastolic blood pressure and faster heart rate and had lower percentage of male patients at presentation. Comorbidities, including hypertension, diabetes, previous coronary artery disease (CAD), previous cerebrovascular accident (CVA) and previous heart failure, were more common in IGFR group but percentage of smoking and family history of CAD were lower (Table 1).

**Table 1:** Baseline characteristics between those with and without IGFR

**Number (%)/Mean (SD)**	**IGFR**	**Non-IGFR**	**All**	**p value**
	**(N = 1226)**	**(N = 1867)**	**(N = 3093)**	
Sex (male)	852 (69.49%)	1568 (83.99%)	2420 (78.24%)	<0.01
Age (year)	70.27 ± 11.71	58.41 ± 12.50	63.11 ± 13.50	<0.01
Blood pressure (mmHg)				
SBP	139.64 ± 36.36	139.08 ± 30.19	139.30 ± 32.75	0.647
DBP	78.75 ± 22.11	83.51 ± 19.84	81.63 ± 20.89	<0.01
Heart rate (beat per minute)	86.26 ± 25.82	79.66 ± 19.39	82.27 ± 22.38	<0.01
Height (cm)	162.27 ± 8.08	165.12 ± 7.54	164.00 ± 7.88	<0.01
Weight (kg)	65.56 ± 12.43	70.34 ± 12.82	68.44 ± 12.88	<0.01
Waist circumference	89.97 ± 10.01	90.64 ± 9.31	90.39 ± 9.57	0.340
Serum creatinine (mg/dL)	2.74 ± 2.59	0.94 ± 0.18	1.65 ± 1.86	<0.01
Dyslipidemia	479 (39.42%)	724 (39.14%)	1203 (39.25%)	0.873
Hypertension	936 (77.23%)	1019 (55.08%)	1955 (63.85%)	<0.01
Diabetes				
Treated	618 (50.78%)	491 (26.45%)	1109 (36.09%)	<0.01
Diet only	44 (7.32%)	59 (12.37%)	103 (9.55%)	<0.01
Smoker				
Current	337 (28.13%)	948 (51.49%)	1285 (42.28%)	<0.01
Former	246 (20.53%)	261 (14.18%)	507 (16.68%)	
Never	615 (51.34%)	632 (34.33%)	1247 (41.03%)	
Family history				
Yes	134 (15.51%)	394 (26.78%)	528 (22.61%)	<0.01
No	730 (84.49%)	1077 (73.22%)	1807 (77.39%)	
Previous coronary artery disease	394 (32.14%)	350 (18.75%)	744 (24.05%)	<0.01
Previous heart failure	118 (9.62%)	48 (2.57%)	166 (5.37%)	<0.01
Previous cerebrovascular accident	169 (13.78%)	113 (6.05%)	282 (9.12%)	<0.01
**In-hospital cardiovascular events**				
Death	39 (3.18%)	11 (0.59%)	50 (1.62%)	<0.01
Re-infarction	13 (1.06%)	11 (0.59%)	24 (0.78%)	0.144
Stroke	5 (0.41%)	7 (0.37%)	12 (0.39%)	0.886
Acute renal failure	54 (4.40%)	6 (0.32%)	60 (1.94%)	<0.01

SBP, systolic blood pressure; DBP, diastolic blood pressure.

### Pharmacological management during admission and at discharge

**Figure 1:**
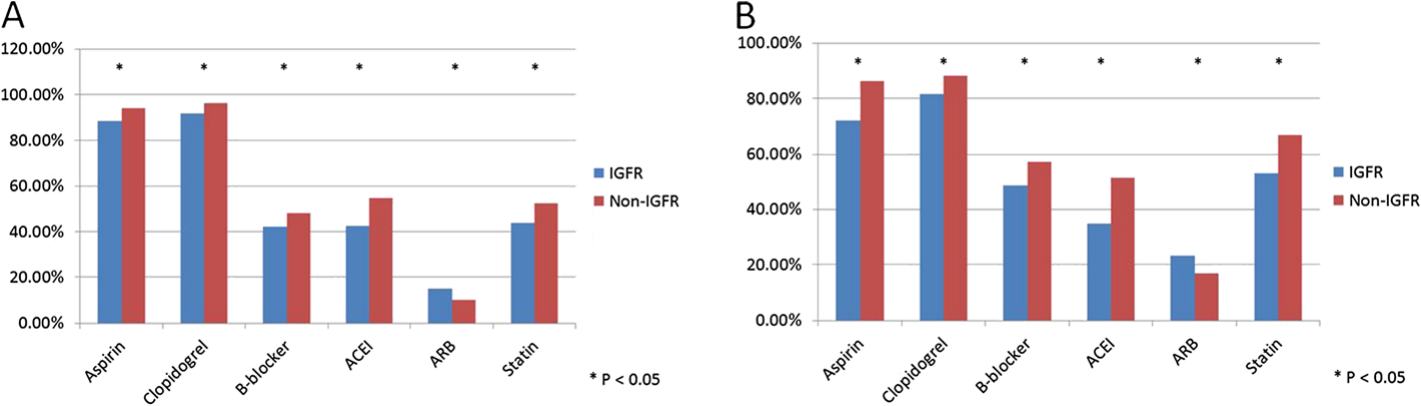
**Medication use (A) during the first 24 hours, (B) at discharge between those with and without IGFR.** IGFR, impaired glomerular filtration rate; ACEI, angiotensin converting enzyme inhibitor; ARB, angiotensin receptor blocker.

Medications prescribed during the first 24 hours and at discharge are shown in Figure 1. Class I guideline-recommended agents including aspirin, clopidogrel, β-blocker, angiotensin converting enzyme inhibitor and statins were significantly less prescribed during admission and at discharge in patients with IGFR than those without IGFR, with the exception of angiotensin receptor antagonist.

### Cardiovascular outcome and IGFR

During admission, the mortality in IGFR patients was significantly higher (3.18% vs 0.59%, p < 0.01) but risks of in-hospital infarction and stroke were similar compared with non-IGFR subjects. In-hospital acute renal failure happened more frequently in the IGFR than non-IGFR patients (4.40% vs. 0.32%, p < 0.01).

**Figure 2:**
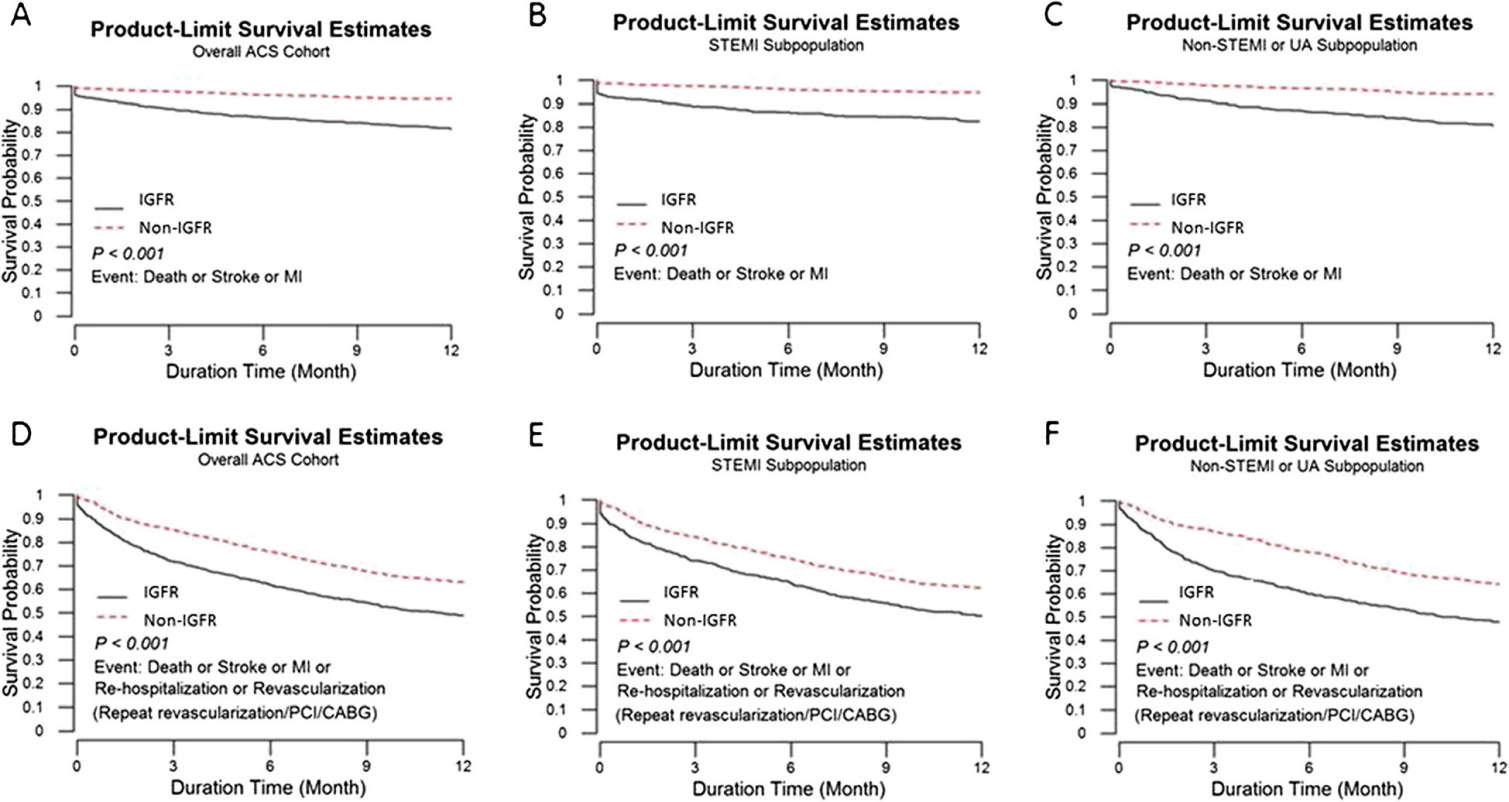
**Kaplan-Meier survival curves of primary and secondary endpoints between those with or without IGFR among the whole (A) and (D), STEMI (B) and (E) or NSTEMI/UA (C) and (F) populations.** IGFR, impaired glomerular filtration rate; STEMI, ST-segment elevation myocardial infarction; NSTEMI, non-ST-segment elevation myocardial infarction; UA, unstable angina; PCI, percutaneous coronary intervention; CABG, coronary artery bypass graft.

Kaplan-Meier survival analysis showed that IGFR was associated with higher primary and secondary outcome for the whole, STE-ACS and NSTE-ACS populations at one year (all p < 0.01) (Figure 2). Cox regression analysis found the adjusted hazard ratio (HR) of presence of IGFR in the whole, STE-ACS and NSTE-ACS populations were 1.98 (95% confidence interval (CI): 1.44-2.73), 1.78 (CI: 1.17-2.72) and 2.27 (CI: 1.38-3.74) for the primary endpoint, respectively (all p < 0.01). For the secondary endpoint, the HR of presence of IGFR in the whole, STE-ACS and NSTE-ACS populations were 1.25 (CI: 1.07-1.45), 1.17 (CI: 0.96-1.44) and 1.33 (CI: 1.06-1.66), respectively.

In the STE-ACS populations the adjusted HRs of the primary and secondary endpoints were 2.30 (95% CI: 1.44-3.68, p < 0.01) and 1.40 (95% CI: 1.10-1.77, p < 0.01) in those with eGFR less than 45 compared with those with eGFR more than 60 ml/min. Compared with those with eGFR > 60 ml/min, the adjusted HRs of the primary and secondary endpoints were 1.27 (95% CI: 0.74-2.19, p = 0.393) and 0.96 (95% CI: 0.74-1.26, p = 0.784) in those with eGFR between 45 and 60 ml/min. For the NSTE-ACS populations the adjusted HRs of the primary and secondary endpoints were 1.24 (95% CI: 0.62-2.47, p = 0.549) and 1.01 (95% CI: 0.74-1.38, p = 0.933) in those with eGFR between 45 and 60 ml/min compared with those with eGFR more than 60 ml/min. Compared with those with eGFR > 60 ml/min, the adjusted HRs of the primary and secondary endpoints were 3.00 (95% CI: 1.77-5.07, p < 0.01) and 1.55 (95% CI: 1.21-1.98, p < 0.01) in those with eGFR less than 45.

### Impact of revascularization strategy and IGFR on cardiovascular outcome

Among 2909 (94.05%) patients undergoing diagnostic angiography, 81.53% were done within 72 hours. Fewer IGFR patients received diagnostic angiography (90.21% vs. 96.57%, p < 0.01) with longer time from admission to diagnostic angiography (51.71 ± 89.97 vs. 31.12 ± 50.04 hours, p < 0.01) compared with non-IGFR subjects. Overall 2628 (85.31%) patients received PCI. IGFR patients had lower percentage of received PCI compared with non-IGFR subjects (79.97% vs. 88.52%, p < 0.01). Nevertheless, the percentage of coronary artery bypass grafting (CABG) in IGFR patients was higher (4.40% vs. 2.68%, p < 0.01).

Among the STE-ACS patients, 84.8% had reperfusion therapy and 96.6% of them received primary PCI as reperfusion strategy. Lower percentage of IGFR patients received reperfusion therapy compared with non-IGFR subjects (80.29% vs. 87.05%, p < 0.01). However, the percentages of primary PCI were similar between two groups (97.48% vs. 97.05%, p = 0.653).

**Figure 3:**
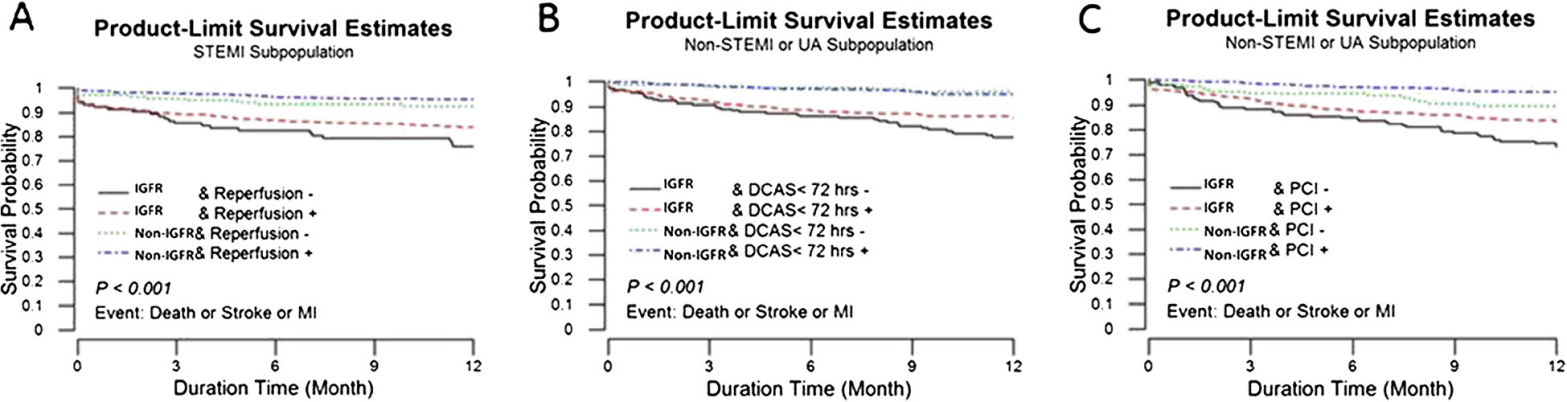
**Interaction between invasive strategy and IGFR on the primary outcome. (A)**. STEMI, **(B)**. NSTEMI or UA and early DCAS, **(C)**. NSTEMI or UA and PCI. IGFR, impaired glomerular filtration rate; STEMI, ST-segment elevation myocardial infarction; NSTEMI, non-ST-segment elevation myocardial infarction; UA, unstable angina; PCI, percutaneous coronary intervention; DCAS, diagnostic coronary angiography strategy.

The Kaplan–Meier survival curves showed significant interaction between revascularization strategy and IGFR on the primary outcome. IGFR patients without invasive strategy had the highest event rate during 1 year follow-up in the whole, STE-ACS and NSTE-ACS populations (all p < 0.01) (Figure 3).

**Figure 4:**
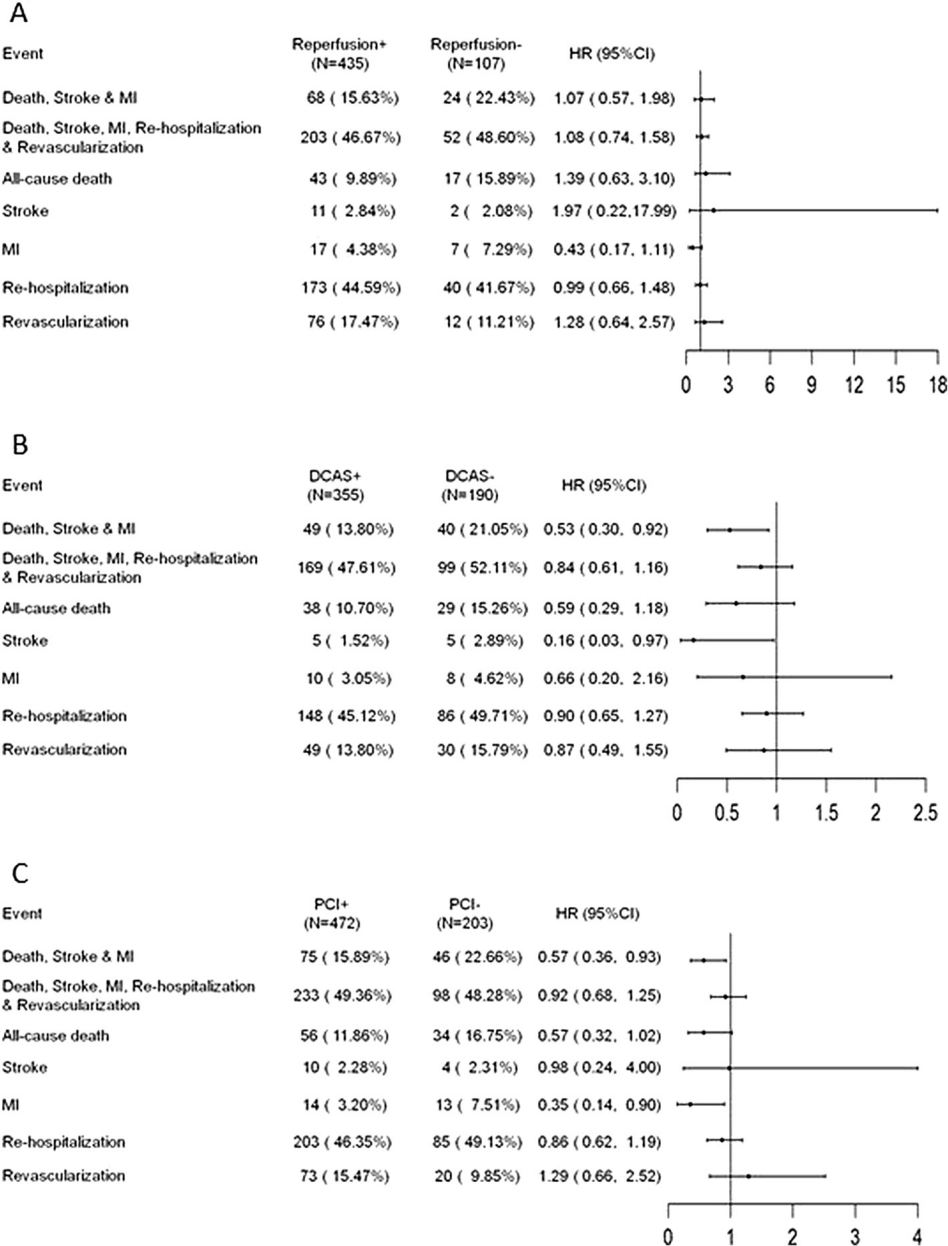
**Effect of invasive strategy on cardiovascular events at 12 months in IGFR population. (A)**. STEMI, **(B)**. NSTEMI or UA and early DCAS, **(C)**. NSTEMI or UA and PCI. IGFR, impaired glomerular filtration rate; STEMI, ST-segment elevation myocardial infarction; NSTEMI, non-ST-segment elevation myocardial infarction; UA, unstable angina; PCI, percutaneous coronary intervention; DCAS, diagnostic coronary angiography strategy.

In IGFR patients Cox regression analysis found invasive strategy did not significantly reduce the primary endpoint (adjusted HR 1.07, 95% CI: 0.57-1.98; p = 0.837) in the STE-ACS (Figure 4A). In the NSTE-ACS population, patients receiving either early DCA only or revascularization had lower primary endpoint as compared with those without invasive strategy (adjusted HR 0.53, 95% CI: 0.30-0.92 and adjusted HR 0.57, 95% CI: 0.36-0.93; p = 0.023 and 0.024, respectively) (Figure 4B and C).

## Discussion and conclusion

There are three major findings in this analysis of Taiwan ACS Full Spectrum cohort study. First, patients with IGFR had more comorbidities but used less guideline-recommended medicines than those without IGFR. Second, ACS patients with IGFR were more likely to suffer from in-hospital death and 1-year cardiovascular events. Third, invasive strategy might be beneficial for the IGFR subjects in the NSTE-ACS population.

### Impaired eGFR and cardiovascular disease

Impaired eGFR is an important poor predictor of prognosis in those with ACS [15,16]. Patients with IGFR have more burden of coronary atherosclerosis in patients with ACS [17]. Although there is no particular reason not to treat IGFR patients just like patients without renal dysfunction, physicians prescribed fewer guideline-recommended treatments even in the absence of contraindications [18]. Furthermore, poor awareness of impaired renal function and its risk in patients with ACS is a big challenge both for the physicians and patients [19]. As shown in our study, those with IGFR had more comorbidities but received fewer guideline-recommended medications. All of above might contribute to the poor prognosis in the ACS patients with IGFR. Therefore, education and prevention strategies are very important both for physicians and IGFR patients such as the nationwide CKD Preventive Project with multidisciplinary care program in Taiwan [4].

### Reperfusion therapy of ST-elevation ACS in IGFR subjects

Underuse of reperfusion therapy is present with all levels of renal dysfunction. Even in patients with mild impairment of eGFR, reperfusion therapy is administered 30% less frequently. The reason for underuse of reperfusion therapy might be due to limited studies investigating the effect of coronary reperfusion. Furthermore, the use of fibrinolysis therapy might be ineffective and even increase the risk of bleeding in the IGFR subjects [20,21]. Therefore, when choosing reperfusion, primary PCI might have greater benefit in patients with IGFR compared with fibrinolytic therapy [22]. As shown in our STE-ACS subgroup, 84.8% received reperfusion therapy and only 3.4% of them received fibrinolysis as reperfusion strategy. However, lower percentage of IGFR patients received reperfusion therapy compared with non-IGFR subjects although the percentages of primary PCI were similar between two groups. Whether primary PCI is superior to fibrinolysis in STE-ACS should be further investigated.

### Early angiography and revascularization in non-ST-elevation ACS

Renal dysfunction is present in one-third of patients with NSTE-ACS [23]. Although there are limited studies evaluating the impact of an invasive strategy on clinical endpoints in patients with NSTE-ACS and IGFR, the European society of cardiology guidelines suggests CKD patients are at high risk of further ischaemic events and therefore should be submitted to invasive evaluation and revascularization whenever possible [24]. PCI is also recommended in patients with CKD amenable to revascularization after careful assessment of the risk–benefit ratio in relation to the severity of renal dysfunction [25]. The 2011 ACCF/AHA guideline suggests invasive strategy is reasonable in patients with mild to moderate (stage II-III) CKD [26]. Our results support the recommendations from current guidelines in providing invasive management in ACS patients with IGFR. Although IGFR patients suffering from ACS had poorer prognosis, early diagnostic angiography with intent to revascularization could improve their cardiovascular outcomes in our study.

### Influence of IGFR and coronary intervention on ACS subjects

Few studies investigated the influence of IGFR on cardiovascular outcomes among ACS population receiving coronary intervention. In patients with STE-ACS receiving primary angioplasty, renal functional impairment based on Cockroft-Gault creatinine clearance or serum creatinine is associated with increased risk of mortality [27,28]. In the New York State data, both STE-ACS and NSTE-ACS patients with IGFR and undergoing PCI have significantly worse in-hospital outcomes [29]. In the GRACE registry primary PCI was associated with lower in-hospital mortality only in patients with normal renal function but no reduction in those with STE-ACS or new-onset of left bundle branch block and renal dysfunction using the MDRD formula [22]. In the SWEDEHEART study early revascularization within 14 days was associated with increased 1-year survival in NSTE-ACS patients with mild to moderate renal impairment, but no association was observed in those with severe or end-stage kidney disease [9]. Hachinohe D. et al. also found early invasive strategy is not beneficial in the severe renal insufficiency population [30]. The present study found IGFR is associated with poor cardiovascular outcome and aggressive strategy given within 72 hours reduced the future occurrence of death, recurrent myocardial infarction and stroke especially in the NSTE-ACS population.

### Limitations

This study has four main limitations. Firstly, it is a nonrandomized and an observational study. Nonetheless, this study provides valuable real-world data on the current practices across the full spectrum of ACS in a CKD endemic area, which could help to improve the ACS management in this population. Second, the procedural details of revascularization strategy are not available. The lesion characteristics, adjunctive medication and device use might modulate the cardiovascular outcome. Third, the renal endpoint is not routinely collected after discharge in this registry. Although those receiving aggressive revascularization treatment had better cardiovascular outcomes, their renal outcome is unclear. However, Inrig et al. previously reported that ACS patients submitted to angiography or angioplasty had no significant long-term decrease in renal function [31]. The authors suggested that the risk of cardiovascular death for patients with IGFR outweighed the risk of renal function loss or development of chronic dialysis, and angiography or PCI should not be contraindicated in this group. Fourth, there is no universal definition of acute renal failure in the registry. The in-hospital acute renal failure was judged at the physicians’ discretion.

In conclusion in this real-word registry we found ACS patients with IGFR received fewer evidence-based medicines although having more comorbidities. Furthermore, they were more likely to suffer from in-hospital and 1-year cardiovascular events. Because coronary revascularization could reduce the 1-year cardiovascular events especially in the NSTE-ACS populations, physician should aggressively treat IGFR population after careful assessment of the risk and benefit.

## Abbreviations

CKD: Chronic kidney disease; ESRD: End-stage renal disease; CVD: Cardiovascular disease; ACS: Acute coronary syndrome; eGFR: Estimated glomerular filtration rate; IGFR: Impaired glomerular filtration rate; PCI: Percutaneous coronary intervention; STE-ACS: ST-elevation acute coronary syndrome; EIS: Early invasive strategy (EIS); NSTE-ACS: Non-ST-elevation patients acute coronary syndrome; DCA: Diagnostic coronary angiography; CAD: Coronary artery disease; CVA: Cerebrovascular accident.

## Competing interest

The authors declare that they have no competing interest.

## Authors’ contributions

THL, HTH, CLW, WTL, AHL, CTK, JJH and FTC conceived of the study, and participated in its design and coordination. THL drafted the manuscript. SCC and CJC performed the statistical analysis. All authors read and approved the final manuscript.

## Pre-publication history

The pre-publication history for this paper can be accessed here:

http://www.biomedcentral.com/1471-2369/15/66/prepub
